# Population genetics and diversity structure of an invasive earthworm in tropical and temperate pastures from Veracruz, Mexico

**DOI:** 10.3897/zookeys.941.49319

**Published:** 2020-06-16

**Authors:** Diana Ortíz-Gamino, Josefat Gregorio, Luis Cunha, Esperanza Martínez-Romero, Carlos Fragoso, Ángel I. Ortíz-Ceballos

**Affiliations:** 1 Instituto de Biotecnología y Ecología Aplicada (INBIOTECA), Universidad Veracruzana. Av. de las Culturas Veracruzanas No. 101, Col. Emiliano Zapata, 91090 Xalapa, Veracruz, México Universidad Veracruzana Xalapa Mexico; 2 Subdirección de Recursos Naturales y Cambio Climático del H. Ayuntamiento de Xalapa, Calle Volcán de Colima 7, Coapexpan 91070, Xalapa, Veracruz, México Subdirección de Recursos Naturales y Cambio Climático del H. Ayuntamiento de Xalapa Xalapa Mexico; 3 Consejo Nacional de Ciencia y Tecnología - Centro de Investigación en Biotecnología Aplicada, Instituto Politécnico Nacional, Av. Insurgentes Sur 1582, Col. Crédito Constructor, Del. Benito Juárez, Ciudad de México, 03940, México Instituto Politécnico Nacional Mexico Mexico; 4 School of Applied Sciences, University of South Wales, Pontypridd, UK University of South Wales Pontypridd United Kingdom; 5 Centro de Ciencias Genómicas, Universidad Nacional Autónoma de México Campus Morelos, Av. Universidad s/n Col. Chamilpa 62210, Cuernavaca, Morelos, México Universidad Nacional Autónoma de México Campus Morelos Cuernavaca Mexico; 6 Instituto de Ecología, A.C., Carretera antigua a Coatepec 351, El Haya 91070, Xalapa, Veracruz, México Instituto de Ecología Xalapa Mexico

**Keywords:** Agroecosystems, asexual reproduction, exotic earthworm, peregrine species, Rhinodrilidae

## Abstract

*Pontoscolex corethrurus* (Müller, 1857) is an invasive tropical earthworm, globally distributed. It reproduces through parthenogenesis, which theoretically results in low genetic diversity. The analysis of the population structure of *P. corethrurus* using molecular markers may significantly contribute to understanding the ecology and reproductive system of this earthworm species. This work assessed the genetic diversity and population structure of *P. corethrurus* with 34 polymorphic inter simple sequence repeat markers, covering four populations in tropical and temperate pastures from Veracruz State. Nuclear markers distinguished two genetic clusters, probably corresponding to two distinct genetic lineages. The number of clones detected in the AC population was lower than expected for a parthenogenetic species. Also, the apparent lack of differences in population structures related to the geographic region among the populations studied may indicate that human-mediated transference is prevalent in these areas. Still, most individuals apparently belong to lineage A, and only a few individuals seem to belong to the lineage B. Thus, the admixture signatures found among the four populations of *P. corethrurus* may have facilitated a successful invasion by directly increasing fitness. In summary, addressing the genetic variation of *P. corethrurus* with ISSR markers was a suitable approach, as it evidenced the genetic diversity and relationships in the populations evaluated.

## Introduction

Earthworms are not only ubiquitous ecological engineers of soil that create biogenic structures; they also sustain the functioning of the ecosystem through their fundamental actions (Lavelle et al. 1997; Brown et al. 2001; Barros et al. 2004). Despite the importance of soil organisms in ecosystem functioning, the soil ecosystem seems to be poorly studied (González et al. 2006). In fact, for a long time, little attention has been given to invasive soil organisms such as earthworms (Gates 1954), despite a rise in belowground invasion over the past 30 years (Hendrix et al. 2002; Craven et al. 2016; Cicconardi et al. 2017). In Mexico, the first records regarding exotic earthworms date from 1900–1906, being lumbricids (*Dendrobaena octaedra* Savigny, 1826; *Lumbricus terrestris* Linnaeus, 1758), megascolecids (*Metaphire californica* Kinberg, 1867), benhaminis (*Dichogaster bolaui* Michaelsen, 1891), and rhinodrilids (*Pontoscolex corethrurus* Müller, 1857) the earthworms registered (Eisen 1900; Michaelsen 1900; Beddard 1912). Since then, 51 exotic species have been described across the country by classical taxonomy (Fragoso and Rojas 2014). Several factors acting at different temporal and spatial scales are involved in earthworm invasion, but the overall picture is not yet understood (Marinissen and Van den Bosch 1992; Baker et al. 2006; Brown et al. 2006; González et al. 2006; Cameron and Bayne 2009; Marichal et al. 2012). In general, the genetic characteristics of invasive organisms have profound impacts on their establishment capacity, range expansions, and successful invasion (Lee 2002).

*Pontoscolex corethrurus*, formerly included in Glossoscolecidae but is now placed in Rhinodrilidae (James 2012), is an earthworm species originating in South America, in the Guiana Shield area of the Amazon (Gates 1954; Righi 1984). The global and local distribution of *P. corethrurus* have been addressed, as well as its interspecific interactions (Ortíz-Gamino et al. 2016; Taheri et al. 2018). It has been hypothesized that the successfulness of *P. corethrurus* is based on its genetic plasticity, which in turn is given by high genomic promiscuity associated to its reproductive strategies (Vitturi et al. 2002; Bengtsson 2009; Fernández et al. 2013; Cunha et al. 2014; Pavlíček et al. 2016).

Parthenogenesis is common in earthworms, usually associated with dispersal, where a single propagule is usually sufficient to stablish a new population (Terhivuo and Saura 2006). Thus, rapid adaptation of parthenogenetic clonal populations may be an essential mechanism for a successful colonization event, as is the case of *P. corethrurus* (Gates 1973; Lavelle et al. 1987; González et al. 2006; Hendrix et al. 2006; Terhivuo and Saura 2006; Buch et al. 2011). Under such a scenario, the genetic variation of *P. corethrurus* in regions far from its natural geographic range may be low if only a single or very few individuals were colonizers (Dupont et al. 2012). Through mitochondrial and nuclear markers, 792 earthworms collected recently over 25 countries demonstrated that *P. corethrurus* is a complex of cryptic species. This is represented by a monophyletic clade composed of four morphologically indistinguishable lineages named as L1, L2, L3, L4 (Taheri et al. 2018).

Around the world, *P. corethrurus* is distributed from 0 to 2000 m a.s.l., with an average altitude of 463 m (Fragoso et al. 1999). In Mexico, specifically in Veracruz State, its distribution ranges from 0 to around 1600 m a.s.l., living at an average temperature of 17 °C (Ortíz-Gamino et al. 2016). The distribution of *P. corethrurus* in Mexico seems to be strongly associated with human-mediated dispersal due to agricultural activities (González et al. 2006; Feijoo et al. 2007; Hendrix et al. 2008; Dupont et al. 2012). Moreover, although Taheri and co-workers have determined recently that *P. corethrurus* living in the State of Veracruz correspond to lineages L1 and L3 (Taheri et al. 2018), this finding was based on only a few specimens, which is likely not representing the entire population. For this reason, further research on population genetics may significantly contribute to understanding the ecology of *P. corethrurus* in Mexico. Thus, the objective of this work was to explore the genetic variation and population structure in *P. corethrurus* inhabiting tropical and temperate pastures in Mexico using a molecular approach based on ISSR markers.

## Materials and methods

### Sampling sites and animal collection

**Figure 1.**
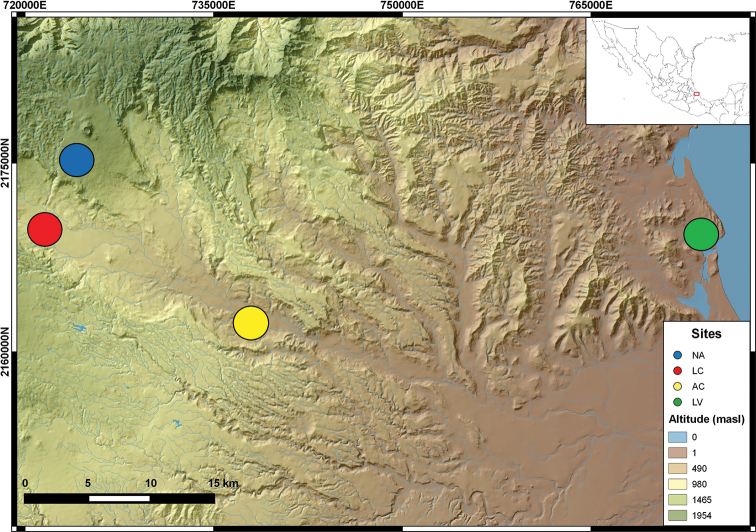
Pastures sampled in the central region of Veracruz State, Mexico. LV, Laguna verde; AC, Actopan; LC, La Concepción; NA, Naolinco. The digital elevation model was created using data provided by Instituto Nacional de Estadística y Geografía, México.

Sampling points were established according to the different attributes of the sites studied in Veracruz State, Mexico (Ortíz-Gamino et al. 2016). In brief, the sampling sites were Laguna Verde (LV), Actopan (AC), Ingenio La Concepción (LC) and Naolinco (NA) (Table 1 and Figure 1), each of them with characteristic ecological attributes. During September 2013, 40 mature (clitellate) individuals of *P. corethrurus* were collected (*N* = 10 per site). Earthworms were kept in plastic boxes with moistened soil and transported to the laboratory at INBIOTECA for taxonomical and anatomical identification (Moreno and Borges 2004). Specimens were rinsed in water to remove soil particles and were fixed with 96% ethanol. All samples were kept at -20 °C until further processing.

**Table 1.** Attributes of earthworms sampling of four pastures in central Veracruz State, Mexico.

Sampling Site	Altitude (m a.s.l.)	Climate	Grass species	Soil texture (%)		
				Clay	Silt	Sand
Laguna Verde (LV)	24	Aw1(w)g	*Paspalum conjugatum*, *Cynodon nlemfuensis*	26.6	28.1	45.3
Actopan (AC)	480	Aw0(w)gw”	*Saccharum officinarum* L.	12.8	32.3	54.9
La Concepción (LC)	973–1036	(A)Ca(f)gw”	*Paspalum conjugatum*, *Cynodon nlemfuensis*	26.6	28.2	45.3
Naolinco (NA)	1566–1667	Cb(fm)gw”	*Paspalum conjugatum*, *Cynodon nlemfuensis*, *Pennisetum clandestinum*	12.8	32.3	54.9

Key: Climate: Aw1(w)g and Aw0(w)gw” are used for warm and sub-humid climate; (A)Ca(f)gw” for warm and humid climate; Cb (fm)gw”, for wet and semi-humid climate. For further details (mean temperature, evapotranspiration, total annual precipitation, etc.), refer to Ortíz-Gamino et al. (2016).

### DNA isolation and quantification

Tail-wall tissue was used for extraction of genomic DNA. Total DNA was extracted using the DNeasy Blood & Tissue kit (Qiagen, Mainz, Germany) following the manufacturer’s instructions. DNA was checked for quality by gel electrophoresis and quantified using a spectrophotometer (ND-2000, Nanodrop Technologies, Wilmington, DE).

### Inter Simple Sequence Repeat (ISSR) protocol

Forty specimens of *P. corethrurus* (*N* = 10 per site) were used for ISSR screening. ISSR screening was based on five primers (Table 2) previously reported to produce polymorphic and reproducible DNA fingerprints for *Eudrilus eugeniae* (Kinberg, 1867) and *Eisenia fetida* (Savigny, 1826) (Sharma et al. 2011). Each PCR reaction contained 1X PCR buffer, 2 mM MgCl_2_, dNTPs at 100 µM each, primers at 0.8 µM each, 1.5 U of Taq DNA polymerase, 1ug/ul BSA and 30 ng template DNA. The PCR reaction mix was brought to a final volume of 10 µL with water. PCR amplifications were performed in an Eppendorf Mastercycler (Eppendorf, Hamburg, Germany) according to the following conditions: an initial step at 95 °C for 3 minutes, followed by 35 denaturation cycles at 95 °C for 30 seconds, annealing at primer-specific temperature for 30 seconds, and elongation at 72 °C for 1 minute. A final extension was performed at 72 °C for 10 minutes. The PCR products were visualized in 2% agarose gels (with ethidium bromide at 1ul/ml). Although the initial screening used a total of 18 primers, only those primers that were polymorphic and reproducible were selected for subsequent analysis (Table 2).

**Table 2.** Primers used for PCR amplification of *Pontoscolex corethrurus* genomic DNA.

Primer	Sequence	Ta (°C)	Maximum number of bands	Estimated size (bp)
840	GAGAGAGAGAGAGAGAYT	59.5	8	2000-200
834	AGAGAGAGAGAGAGAGYT	61	7	2000-300
866	CTCCTCCTCCTCCTCCTC	70	6	2000-400
810	GAGAGAGAGAGAGAGAT	52.4	6	2000-400
807	AGAGAGAGAGAGAGAGT	54.4	7	2000-300

### Data analysis

The amplified DNA fragments were transformed into a binary matrix (1 = presence, 0 = absence), as reported previously (Abbot 2001). A multilocus genotype (MLG) was constructed for each individual by pooling data of single ISSR fingerprints using the procedure available in the POPPR package in R for genetic analysis of populations (Kamvar et al. 2014). Isolates with the same MLG were considered clones, and some analyses were conducted for the original and clone-corrected datasets (Gramaje et al. 2014). The POPPR package in R was used to calculate dissimilarity distance matrices and generate a minimum spanning network from these matrices (Kamvar et al. 2014). To assess the potential evolutionary relationships among MLGs, a minimum spanning network was constructed using the genotypes of earthworm from each sampling location. Bootstrapping was performed with 1000 bootstrap resampling. Genotypic diversity, genetic richness and the evenness index were calculated for each population. The ‘rarecurve’ function from the VEGAN package in R (Oksanen et al. 2007) was used to generate rarefaction curves. Curves were calculated to determine whether the sampling intensity was adequate to detect most of the MGLs in each *P. corethrurus* population. Additionally, the minimum number of loci needed to distinguish all MGLs was also calculated. Given that sample size varied among populations, we employed rarefaction to explore the effect of sample size on observed MLGs.

### Genetic differentiation, structure, and clustering analyses

The genetic variance for all MLGs was estimated through an analysis of molecular variance (AMOVA) using the GenAlEx v.6.5 software (Peakall and Smouse 2006). Genetic variance relative to total variance was calculated as PhiPT (analog of the F_st_ fixation index) for all populations, as well as regarding within-population genetic variance (Peakall and Smouse 2006). The significance was computed by using 9,999 permutations, and the confidence interval at 95%, by 10,000 re-samplings. For this analysis, only single copies of the different genotypes were used to give identical weight to MLGs. The Mantel test was used to explore the potential correlation between the matrix of genetic differentiation between pairs of MLGs and the matrix of spatial distances between populations, using Arlequin v.3.5 (Excoffier and Lischer 2010). The association between *P. corethrurus* individuals was assessed initially using a Principal Components Analysis (PCA) implemented in GenAlEx v. 6.51 (Peakall and Smouse 2006). As PCA is independent of any genetic hypotheses it is suitable for the analysis of partially clonal species. Additionally, Unweighted Pair Group Method with Arithmetic Mean (UPGMA) dendrograms were also created using the POPPR package (Kamvar et al. 2014). Bootstrapping was performed with the PVCLUST package in R using 10,000 bootstrap re-samplings (Suzuki and Shimodaira 2006).

Population structure was explored using the Bayesian clustering method implemented in STRUCTURE v.2.3.4 (Pritchard et al. 2000), as well as a distance-based approach using a discriminant analysis of principal components (DAPC) (Jombart et al. 2010). STRUCTURE v.2.3.4 was used to identify the number of genetic clusters within the dataset, and to assign individuals to the clusters defined using an admixture model. To this end and to confirm consistency, 15 replicate runs were carried out for each K (1–8). The most likely value of K was determined with “BestK” implemented in CLUMPAK (Evanno et al. 2005), which uses the ΔK method of Evanno et al. (2005). The results from the 15 replicate runs were pooled using CLUMPAK online version (Evanno et al. 2005). Relative dissimilarity distances were calculated according to the index of association (Brown et al. 1980; Smith et al. 1993). The approach returns a distance reflecting a ratio of the number of observed differences to the number of possible differences.

The hybridization status of individuals according to the Bayesian genetic clusters defined in Structure (defined as putative Lineage A and Lineage B) was further investigated using NEWHYBRIDS v1.1 (Anderson and Thompson 2002), which also uses a Bayesian assignment by implementing a multilocus allele frequency model-based approach. This approach clusters together MGLs without *a-priori* knowledge of parental allele frequencies, and also has the advantage of specifically assuming a mixture of parental and several hybrid classes (F1’s, F2’s, and various backcrosses as B1 and B2 hybrids) to assign them into categories. Individual posterior probabilities belonging to each hybrid category were estimated using the MCMC method in a Bayesian framework using Jeffreys-type priors and a burn-in period of 100000 iterations followed by 50000 sweeps from the posterior distribution sampling (Anderson and Thompson 2002). Linkage disequilibrium as an indication of random mating was calculated and tested for significance with 1,000 randomizations using the POPPR package in R (R core team 2004). The measures of gametic disequilibrium tested were the index of association (*I*_A_) (Brown et al. 1980; Smith et al. 1993) and a standardized alternative of the *I*_A_ (r̄_d_) (Agapow and Burt 2001). The null hypothesis for this test is that there is a random association among alleles at different loci and *I*_A_ = 0; the null hypothesis for random mating is rejected where if *I*_A_>0.

## Results

Beyond the ecological relevance of *P. corethrurus*, information on genetic variability is relevant to determine the selective forces that act on the reproductive system of this species. In that sense, the survey carried out in this study was aimed to reveal the population genetic structure of *P. corethrurus* in natural landscapes covering tropical and temperate pastures. For this, a set of ISSR markers were used to assess the genetic diversity and population structure of *P. corethrurus* genotypes from four locations in Veracruz State, Mexico.

### Genotypic diversity of *P. corethrurus* populations

**Figure 2.**
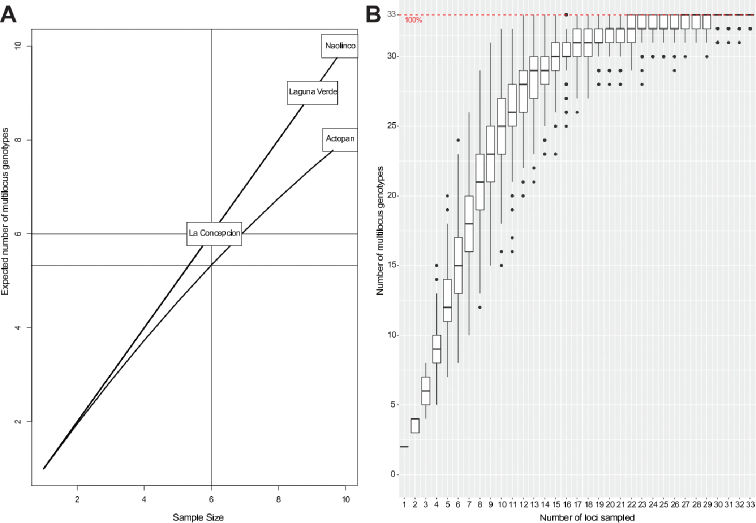
Rarefaction curve of expected number of MLGs captured per earthworm of *Pontoscolex corethrurus* sampled (**A**), and a MLG accumulation curve according to the number of loci sampled (**B**).

Following a PCR-based approach, ISSR primers produced bands on agarose gel that were suitable for assessing the genetic diversity and genetic relationship between and across populations of *P. corethrurus*. The PCR products ranged between ~200 and ~2000 bp from genomic DNAs of *P. corethrurus*. The total number of bands and polymorphism rates are shown in Table 3. A total of 33 MLGs among the 35 *P. corethrurus* individuals yielded reliable products. Overall, one MLG corresponding to AC was observed twice, whereas the rest of MLGs (31) were detected only once. As regards MLG diversity (H), this parameter varied across populations, with no correlation to any specific geographic location (Table 3). On the other hand, in contrast to AC, evenness values (E_5_) were higher for LV, LC, and NA, in agreement with the fact that all genotypes found were unique to these populations (Table 3). Nei’s unbiased gene diversity (H_exp_) values varied from the highest (LC = 0.39) to the lowest (NA = 0.29). Most populations exhibit low genetic diversity (Table 3), except for the NA population, which displays a higher genetic diversity. This was supported by the rarefaction curves, which indicated that NA had a higher number of sampled loci, as well as higher MGLs, than the other three populations (Figure 2A). Interestingly, the minimum number of loci needed to define the total number of MLGs found reached a plateau after 18 loci (Figure 2B). Moreover, the variation among and within populations assessed with AMOVA resulted in values of 25% and 75%, respectively (Table 4). Altogether, all populations showed high genotypic diversity, and according to the PhiPT value (analog of F_st_ fixation index), there were significant differences between the LV, AC, and NA populations were found (Table 4).

**Table 3.** Parameters of genetic variation in four *Pontoscolex corethrurus* populations living in central Veracruz State, Mexico.

Sampling Site	N	MLG	eMLG	Pb	Tb	H	G	*E*_5_	H_exp_	Ia	rbarD
**LV**	9	9	9	20	34	2.20	9	1	0.30	1.98*	0.10*
**AC**	10	8	8	22	34	2.03	7.14	0.93	0.30	3.23*	0.15*
**LC**	6	6	6	27	34	1.79	6	1	0.39	1.16*	0.04*
**NA**	10	10	10	24	34	2.30	10	1	0.29	4.08*	0.18*
Total	35	33	9.85			3.48	31.41	0.97	0.40	1.65	0.05

Abbreviations: N, samples; MLG, Multilocus genotypes; eMLG, estimated MLG; H, genetic diversity; G, Evenness index; E_5_, evenness values, Hexp, Nei’s unbiased gene diversity; Ia, Index of Association; rbarD, standardized index of association.

**Table 4.** Analysis of molecular variance (AMOVA) testing for genetic differentiation between four populations of *Pontoscolex corethrurus* living in central Veracruz State, Mexico (A), and PhiPT pairwise comparisons (B).

	Level of variation	d.f.	SS	MS	Est. Var.	Proportion (%)
A)	Among Populations	3	59.079	19.693	1.760	25%
	Within Populations	29	154.497	5.327	5.327	75%
	Total	32	213.576		7.088	100%
	**Population**	**Laguna Verde**	**Actopan**	**La Concepcion**	**Naolinco**	
B)	Laguna Verde	0.000				
	Actopan	0.229**	0.000			
	La Concepcion	0.069	0.161**	0.000		
	Naolinco	0.335**	0.347**	0.199*	0.000	

Abbreviations: d.f., degrees of freedom; SS, sum of squares; MS, mean squares; Est. Var., estimated variance; %, proportion of molecular variation. Significance levels as follows: * p < 0.05 and ** p < 0.01.

### Clustering of MLGs: relationships between- and within *P. corethrurus* populations

**Figure 3.**
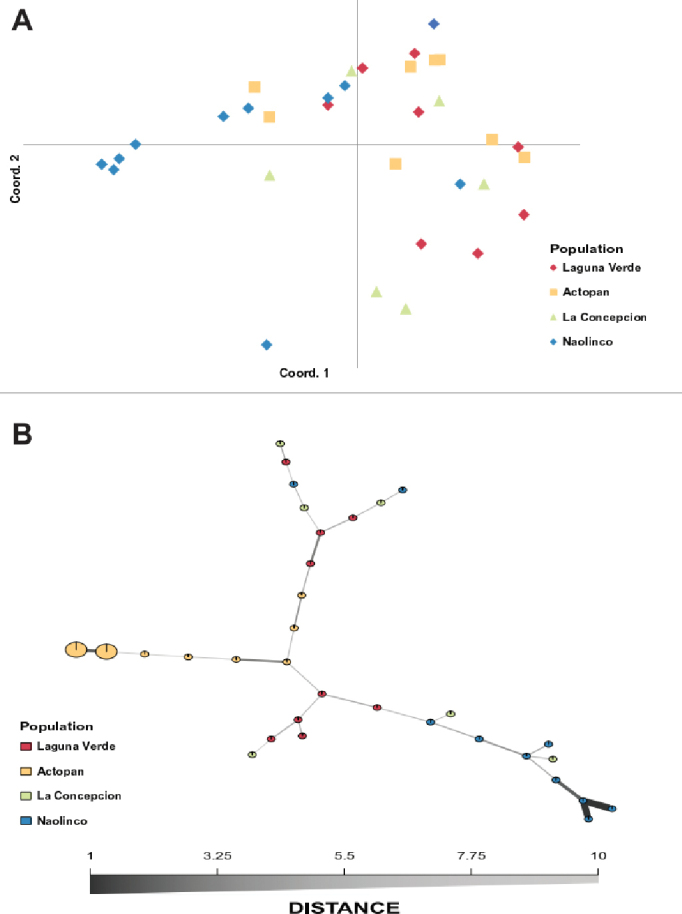
A Principal Components Analysis, where colors indicate specimens of the population (**A**) and a Minimum Spanning Network where each node denotes a different MLG, with size matching the number of individuals. Edge thickness and color are proportional to absolute genetic distance. Edge lengths are arbitrary (**B**). Both analyses show the relationship between multilocus genotypes (MLGs) for four different earthworm populations of *Pontoscolex corethrurus* living in central Veracruz State, Mexico.

**Figure 4.**
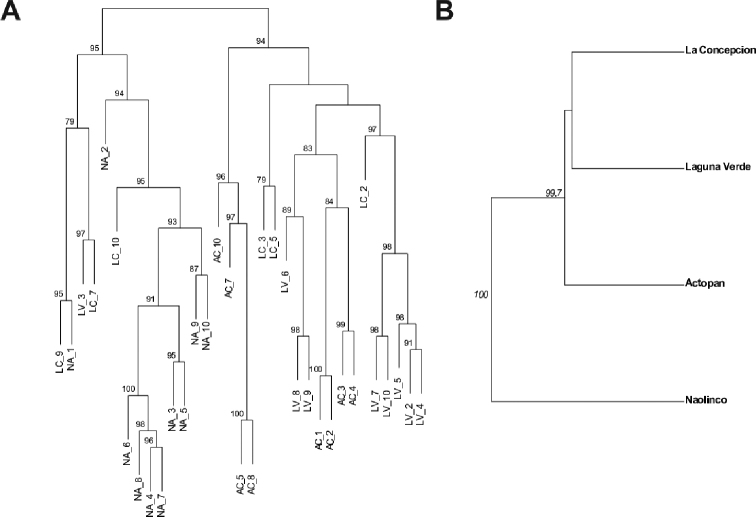
UPGMA dendrogram of genetic distance between MGLs (**A**) and between populations (**B**) observed in the distinct populations of *Pontoscolex corethrurus* collected in central Veracruz State, Mexico. Only bootstrap values higher than or equal to 70% are shown.

Although some individuals cluster together according to site (e.g., animals in NA), most of the individuals scattered in a non-uniform clustering (Figure 3A). As shown in Figure 3A, axes 1 and 2 of the PCA scatter plot accounted for 26% and 15% of total genetic variability, respectively. On the other hand, the global minimum spanning network showed that all populations have MLGs that are closely related (Figure 3B). The comparison of the matrix of Euclidian genetic distance with the matrix of geographic distances using the Mantel test showed that there is no correlation between these two matrices. Thus, data in the genetic distance matrix is not explained by the geographic positioning of the populations (Figure 3). Moreover, the genetic distance between MLGs and between populations indicate no evident correlation with geographic locations, even though LC and LV appeared to be genetically related (Figure 4A, B, respectively). In summary, the clustering analysis shows no clear association with geographic distances.

### Estimation of population structure according to genetic clusters

**Figure 5.**
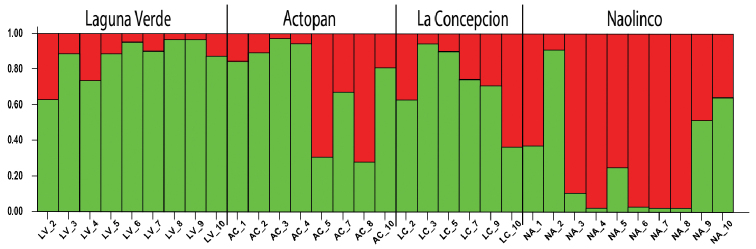
Estimated population genetic structure with a summary plot of Q estimates based on the ISSR data observed for four populations of *Pontoscolex corethrurus* in central Veracruz State, Mexico. Each individual is shown by a vertical line, which is partitioned into colored segments representing the fraction of the number of members in cluster K (%).

**Figure 6.**
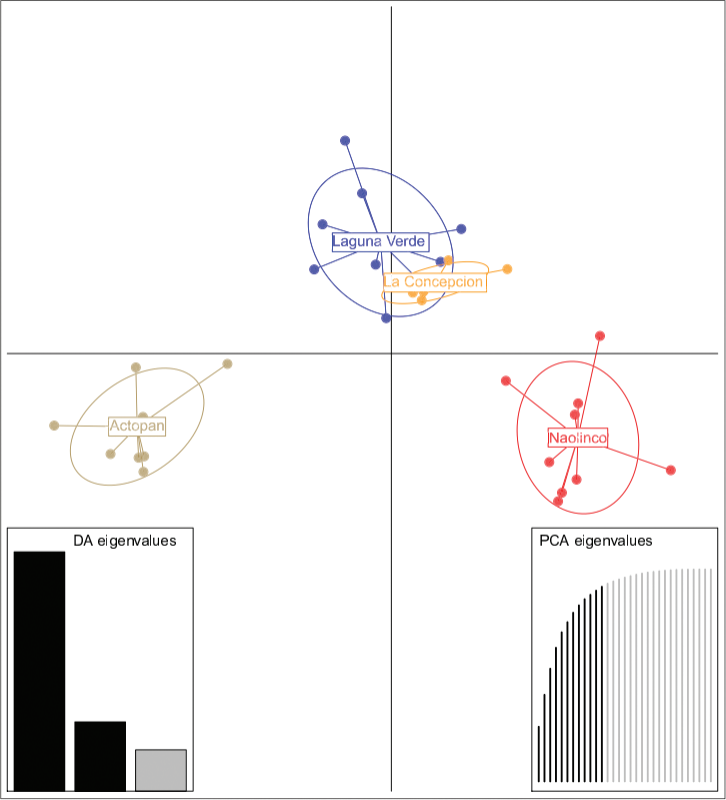
Genetic structure using ISSR data for 35 *Pontoscolex corethrurus* individuals based on discriminant analysis of principal components (DAPC). Proportion of eigenvalues in discriminant analysis (bottom left plot) and PCA eigenvalues (bottom right), with the first 12 significant principal components highlighted in black.

**Figure 7.**
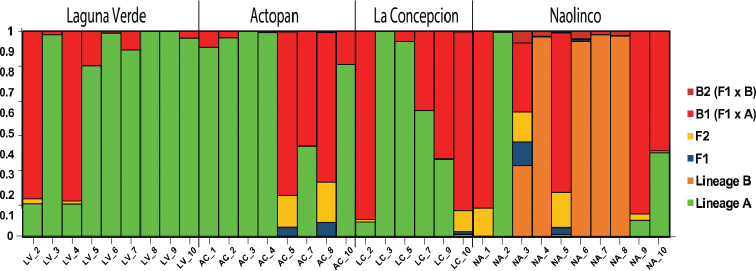
Classification of *Pontoscolex corethrurus* individuals according to a Bayesian assignment algorithm implemented in NEWHYBRIDS (Anderson and Thompson 2002) to detect gene flow. Each unit represents an individual corresponding to parental lineages (Lineage A and Lineage B), F1 generation, F2 (F1 x F1) and later generation or introgressive hybrids B1 (Lineage A x F1) and B2 (e.g., Lineage B x F1).

The Bayesian analysis of population structure estimated two distinct genetic clusters (K = 2, Ln P (D) = -557.89 ± 0.31) distributed across the four geographic locations (Figure 5). Similar to the results obtained by the PCA and dendrogram analyses, the Bayesian analysis revealed no apparent structure that could be associated with geographic location (Figure 5). Nevertheless, the NA population seems to belong mostly to cluster 2 and the one from LV to cluster 1, whereas AC and LC appear to be strongly admixed (Figure 5). From this analysis, two distinct genetic lineages can be identified among populations, henceforth defined as lineages A (mostly LV individuals) and lineage B (mostly NA individuals). Besides, the DAPC analysis confirmed the above genetic relationship between LC and LV, even when they belong to distant geographic locations (Figure 6). On the other hand, similar to the results of the Bayesian analysis, AC (lineage A) and NA (lineage B) are detached (Figure 7). Notably, both clone-corrected (*N* = 33) and uncorrected (*N* = 35) reject the hypothesis of no linkage among markers (Table 3), supporting asexuality in all populations (Suppl. material 1: Figure S1). Altogether, the assessment of genetic diversity in the four populations of *P. corethrurus* suggests that they belong to two different lineages, with some relationships among them despite their distribution in different locations.

## Discussion

Soil texture and chemical composition are among the key environmental variables that play a role in the invasion process of earthworms (Hendrix and Bohlen 2006; Marichal et al. 2012; Craven et al. 2016). On the other hand, traits intrinsic to this species, such as dispersal capacity, life history, and reproduction mode, are known to influence the genetic structure of earthworms (González et al. 2006; Cameron et al. 2008; Marichal et al. 2012). All these features can contribute to the isolation of populations, favoring intra-specific relationships that determine the population structure (Baker 1998; Novo et al. 2010; Fernández et al. 2013). Thus, determining the genetic variability in asexual organisms, such as the earthworm *P. corethrurus*, is of interest, since it may influence population structure and therefore, putative evolutionary bottlenecks.

In this work, the study of genetic diversity and population structure of four populations living in Mexican tropical and temperate pasture was carried out through a molecular approach using ISSR markers. The use of ISSR markers is well supported by several studies since it produces a high percentage of polymorphic loci (Abbot 2001; Dušinský et al. 2006; Luan et al. 2006; Sharma et al. 2011; Seyedimoradi and Talebi 2014; Ng and Tan 2015; Buczkowska et al. 2016). Also, ISSR markers potentially discriminate isolated populations by geographic conditions and are able to differentiate cryptic species ((Dušinský et al. 2006; Buczkowska et al. 2016). Finally, since ISSR markers only amplify nuclear regions of eukaryotic genomes, it avoids the amplification of bacterial DNA fragments, i.e., members of the phylum Bacteroidetes, commonly associated with *P. corethrurus* (Bernard et al. 2012; Seyedimoradi and Talebi 2014).

According to PhiPT values, there were significant differences between the LV, AC, and NA populations, meaning that most populations possess high genotypic diversity (Figure 2 and Table 4). In this regard, the AMOVA analyses showed that most genetic diversity (75%) was found within populations, and only 25% among populations. This finding is similar to those reported by Cameron et al. (2008) and Cunha et al. (2014) for *Dendrobaena octaedra* and *P. corethrurus*, respectively. In both cases, most of the genetic variability was found within populations, and only a minor proportion occurred between populations (Cameron et al. 2008; Cunha et al. 2014). The high levels of genotypic diversity observed in this study could be expected because there is evidence of high diversity in populations of *P. corethrurus* using AFLPs (Cunha et al. 2014). It is also likely that the high genotypic diversity observed in the populations of *P. corethrurus* studied is due to the high evolutionary rate of change within ISSR regions compared to other nuclear regions tackled by AFLPs, RAPD or URPs (Sharma et al. 2011; Gramaje et al. 2014; Seyedimoradi and Talebi 2014; Ng and Tan 2015).

As regards the movement of MLGs across the different sites, a conspicuous behavior was observed, particularly in LV, AC, and LC. Such observation may be explained only by an intense human-mediated transference of *P. corethrurus* through road networks or activities associated with agriculture (Lapied and Lavelle 2003; Baker et al. 2006; González et al. 2006; Feijoo et al. 2007; Dupont et al. 2012; Ortíz-Gamino et al. 2016). On the other hand, clustering analyses through PCA and a dendrogram identified a significant admixture across all populations; however, it was possible to identify at least two divergent and well-differentiated genetic clusters (lineages A and B). It is likely that the lineages identified potentially correspond to those previously reported by Taheri and co-workers, namely lineages L1 and L3 (Taheri et al. 2018). It is important to highlight that both our sampling sites and the molecular approach, are different from the work done by Taheri et al. (2018). Their research involved specimens collected in different years, covering an extended period (from 1996 to 2016; Taheri et al. 2018, Suppl. material 1: Figure S1). Despite these differences, our results are consistent with those reported by Taheri et al. (2018), namely, the identification of two lineages in the *P. corethrurus* populations.

Lineage A seems to be widespread, covering LC and LV sites (Figure 6). Such distribution may indicate wide ecological tolerance, with populations probably well adapted to warm temperatures and poor soils. The distribution of lineage A may suggest that this lineage corresponds to L1 (Taheri et al. 2018), since it was found in most of the places sampled. Moreover, our results are similar to those reported for other species, such as *Octolasion tyrtaeum* (Savigny, 1826), for which a single haplotype was found in all sampling stations (Terhivuo and Saura 1993). Hence, for peregrine species, the evidence indicates that human activities are strongly shaping the dispersal pattern of MLGs through incidental transfer in crops soil or fishing (Cameron et al. 2008; Dupont et al. 2012; Ortíz-Gamino et al. 2016). However, there are also reports of the intentional introduction of earthworm species for commercial applications like waste management and land bioremediation (Hendrix 2006).

In contrast to lineage A, lineage B (mostly NA specimens) showed the best distinct cluster of individuals (Figure 5). This cluster is likely associated with the contrasting environmental conditions that predominate in NA, namely higher altitude, three types of grass (*Paspalum conjugatum* P.J. Bergius, *Cynodon nlemfuensis* Vanderyst, *Pennisetum clandestinum* Hochst. ex Chiov.), soil rich in organic matter, or even interactions with other soil organisms (Ortíz-Gamino et al. 2016). All these characteristics could be acting as an environmental screen that results in the clustering of NA individuals (Figure 3). Remarkably, a similar finding has been reported for *Aporrectodea trapezoides* (Dugés, 1828), in which clonal lineages seem to remain close to their original areas, indicative of some level of local adaptation or strong interspecific relationships (Fernández et al. 2013). Thus, the lineage described as L2 by Taheri et al. (2018) could correspond to lineage B in this work. If so, this lineage is likely well-adapted to micro conditions including habitat, feeding habits and biotic interactions. As proposed earlier, another barrier to the dispersal of lineage B may be temperature (Janzen 1967). This could be the case for the NA population, in which the mean annual temperature (17 °C) may be acting as the main barrier to MGL dispersion (Ortíz-Gamino et al. 2016). Importantly, under a global-change scenario, namely intensive land-use change and alarming global warming, this barrier could become weaker, thus enabling the invasion of pantropical earthworm species (Jiménez and Decaëns 2000; Eisenhauer et al. 2014; Gutiérrez and Cardona 2014).

On the other hand, sexual reproduction is a rare event in *P. corethrurus* (Gates 1973), as it is widely accepted that its reproduction occurs mainly by parthenogenesis (Gates 1973). The standardized index of association (r̄ d) supported the hypothesis of clonal population structure (Suppl. material 1: Figure S1). In this sense, r̄ d values suggest a widespread dispersal of MLGs across populations. This contrasts with the linkage disequilibrium tests, in which the null hypothesis of random mating was rejected for all populations. Nevertheless, these results should be interpreted with caution, as it is challenging to demonstrate the presence of linkage disequilibrium with small sample sizes (Hagenblad et al. 2006; Du et al. 2007; Gramaje et al. 2014). In earthworms, parthenogenesis has been associated with polyploidy, as well as with high levels of DNA methylation (Regev et al. 1998). Therefore, it is also plausible that methylation may be fostering the epigenetic control of phenotypic plasticity, which could be crucial for a successful colonization (Stürzenbaum et al. 2009). It is tempting to claim that temperature affects *P. corethrurus* and impacts its reproduction rate, which also may be regulated by polyploidy and epigenetic control (Ortíz-Gamino et al. 2016). Further studies regarding the number of chromosomes or genomic rearrangements are needed to address whether or not these features are linked to environmental features.

In summary, the screening of genetic diversity is helpful to monitor the dynamics of population structure and its relationships to ecological and environmental features and to contribute valuable information about the isolation of invasive earthworm species. In this sense, our work provides evidence of the existence of two lineages of *P. corethrurus* in Veracruz State, Mexico, showing different distribution patterns according to the prevailing environmental conditions found in regions studied. Therefore, our data represent an relevant contribution to know the movement dynamics and diversification of *P. corethrurus*, which will be useful information for planning successful strategies aimed to control or prevent the biological invasion of this species in Mexico.

## Conclusions

Despite being random, biological invasions are intriguing events, mainly because they involve populations of organisms with certain features and particular habits. Intriguingly, a parthenogenic species such as *P. corethrurus* has been successful in colonizing areas all over the world. The interaction of *P. corethrurus* genetics with the environment should drive its selection and distribution pattern.

In this work, we assessed populations of *P. corethrurus* inhabiting tropical and temperate pastures of Veracruz, Mexico, in terms of genetic variation through ISSR markers. Our results revealed the existence of at least two well-differentiated genetic clusters, corresponding to different lineages (lineages A and B). The lineages identified in this work likely correspond to lineages L1 and L3 identified previously by Taheri et al. (2018). Although further research is needed to discern why *P. corethrurus* populations occur in some sites but not in others, our work suggests that genetic variation is playing a key role in the invasion process. The association between the genetic variability of *P. corethrurus* and its success in invading new sites is counter-intuitive due to its parthenogenic reproduction, i.e., clonal multiplication. Additional genotyping of *P. corethrurus* individuals inhabiting diverse environments or different States like Tabasco, Puebla, and Tamaulipas, will be necessary, not only to confirm our results, but to track the dynamics of dispersal and diversification of lineages, as well as to identify new dominant genotypes or newly introduced lineages. All this information should be gathered before using *P. corethrurus* in biotechnology research, remediation, or fishing, or before estimating its effects on local crops, plants, or organisms.

## Supplementary materials

Supplementary material 1 Figure S1 Data type image Explanation note Linkage disequilibrium test using r̄ d as implemented in R package Poppr (Kamvar et al. 2014). Visualizations of tests for linkage disequilibrium, where observed values (blue dashed lines) of r̄ d are compared to histograms showing results of 999 permutations.. https://binary.pensoft.net/file/421629This dataset is made available under the Open Database License (http://opendatacommons.org/licenses/odbl/1.0/). The Open Database License (ODbL) is a license agreement intended to allow users to freely share, modify, and use this Dataset while maintaining this same freedom for others, provided that the original source and author(s) are credited.Diana Ortíz-Gamino, Josefat Gregorio, Luis Cunha, Esperanza Martínez-Romero, Carlos Fragoso, Ángel I. Ortíz-Ceballos
