# Supplementary material for: Population genetics and diversity structure of an invasive earthworm in tropical and temperate pastures from Veracruz, Mexico
Source: Zookeys. 2020 Jun 16;941:49–69. doi: 10.3897/zookeys.941.49319 (PMC7311532; doi:10.3897/zookeys.941.49319)

Supplementary Figure 1. Linkage disequilibrium test using r̄d as implemented in R package Poppr (Kamvar et al., 2014). Visualizations of tests for linkage disequilibrium, where observed values (blue dashed lines) of r̄d are compared to histograms showing results of 999 permutations.


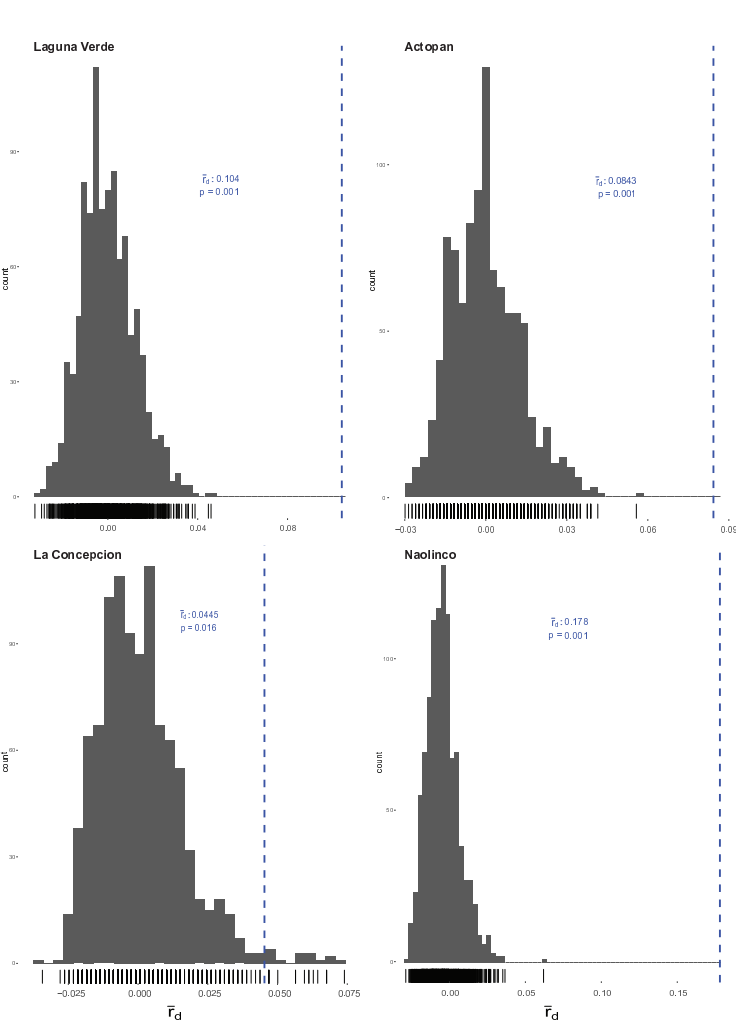

Supplement: Supplementary material 1 — Figure S1 [file zookeys-941-049-s001.docx]
